# Pseudoaneurysm of graft–graft anastomosis of a hand-sewn branched graft: a case report

**DOI:** 10.1186/s13019-015-0356-0

**Published:** 2015-11-05

**Authors:** Toshihito Gomibuchi, Tamaki Takano, Yuko Wada, Takamitsu Terasaki, Tatsuichiro Seto, Daisuke Fukui

**Affiliations:** Department of Cardiovascular Surgery, Shinshu University School of Medicine, Asahi 3-1-1, Matsumoto, Nagano 390-8621 Japan

**Keywords:** Pseudoaneurysm, Bleeding, Aorta operation

## Abstract

**Background:**

Pseudoaneurysm of graft–graft anastomosis is an extremely rare but potentially fatal complication after thoracic aorta replacement with a prosthetic graft. We report a case of pseudoaneurysm at the graft–graft anastomosis of a hand-sewn branched graft.

**Case Presentation:**

A 65-year-old man underwent total arch replacement with a hand-sewn branched graft for Stanford type A acute aortic dissection 22 years ago. During follow-up, serial CT scans showed a pseudoaneurysm on the branched graft which warranted reintervention. Surgical repair involved direct suture of multiple bleeding points which were found at the sites of the hand-sewn branches anastomosis. The postoperative course was uneventful, and no signs of bleeding were observed by CT after the reoperation.

**Conclusions:**

Long-term follow-up is essential to detect late complications at the site of hand-sewn anastomosis.

## Background

Pseudoaneurysm of graft–graft anastomosis is an extremely rare but potentially fatal complication after thoracic aorta replacement with a prosthetic graft, although it is relatively more between the native aorta and a prosthetic graft. We describe a case of pseudoaneurysm at the graft–graft anastomosis of a hand-sewn branched graft 22 years after total arch replacement.

## Case presentation

**Fig. 1:**
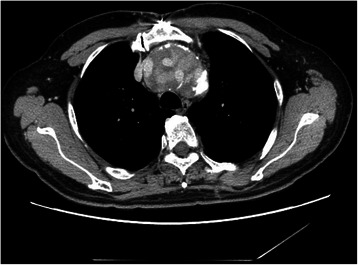
Dye stains are found inside the pseudoaneurysm of the aortic arch

A 65-year-old man had undergone total arch replacement with a Gealseal knitted Dacron graft (Sulzer Vascutek, Scotland, UK) for type A acute aortic dissection 22 years ago. A hand-sewn branched graft consisting of straight grafts 28 mm and 8 mm in diameter was used for the aortic arch. We made three holes on the 28 mm straight graft with a thermal cautery, and three straight grafts of 8 mm diameter were individually sewn with 3–0 polyester sutures before surgery. A 24-mm straight graft was used for the ascending aorta, and the branched and straight grafts were sewn after reconstruction of neck arteries. The patient was discharged from the hospital on postoperative day (POD) 33. He underwent descending aorta replacement six years after the initial surgery and thoracoabdominal aorta replacement 20 years after the initial surgery for enlargement of the redidual dissection. Computed tomography (CT) angiography showed a pseudoaneurysm of 46 mm in diameter on the ascending aorta graft before thoracoabdominal replacement. Aortography was performed but did not reveal the bleeding point; therefore, only a thoracoabdominal aorta replacement was performed. The size of the pseudoaneurysm gradually increased and reached 60 mm, and a dye stain was found inside the pseudoaneurysm after two years of follow up in an outpatient clinic (Fig. 1).

**Fig. 2:**
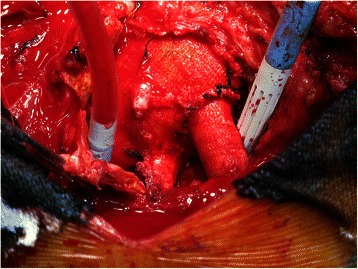
Intraoperative view

We performed median resternotomy after exposing the left femoral and right axillary arteries. Cardiopulmonary bypass (CPB) was established with cannulation of the right axillary artery, the left femoral artery, and the right atrium under normothermia. The pseudoaneurysm was opened and a thrombus inside the aneurysm was removed. Small bleedings were found on the anastomosis of the arch graft–brachiocephalic branch graft (Fig. 2), arch graft–left subclavian branch graft, and ascending graft–arch graft. The bleedings were repaired with interrupted pledget-supported sutures. CPB was weaned off after the other anastomoses including native artery–graft anastomoses were carefully examined. CPB time was 89 min, and total operation time was 427 min.

No postoperative complications or neurological sequelae were observed. A CT angiogram showed no blood leakage on POD 13, and the patient was discharged from the hospital on POD 16.

## Discussion

Pseudoaneurysm of a graft–graft anastomosis long after thoracic replacement is extremely rare, and we could find no previous reports. It has been our practice to realize branched graft for the aortic arch by sewing small diameter grafts to a large diameter graft until the branched graft became commercially available. The Gelseal graft is a gelatin-sealed knitted Dacron graft and has been reported to dilate over time, but graft failure has not been reported [1]. In the present case, the Dacron graft had been implanted for > 20 years and may have resulted in endothelialization and pannus ingrowth in the flow lumen. In an animal study, the average pannus extension from the native aorta to the graft increased from 1–1.5 mm at seven days to 1–2 mm at 10 days and to 2–4 mm at 14 days after implantation in the descending thoracic aorta of a dog. On the 7- and 10-day grafts, 0–5 islands of endothelial-like cells were also found [2]. In another study, the flow surfaces were covered with uniform thin and glistening tissue and complete endothelial-like cell coverage eight weeks after implantation in the thoracic and abdominal aorta of a dog [3]. These studies suggest that healing of the graft progresses over time. However, the luminal surface of the hand-sewn branched graft may not have been covered with patient tissue 20 years after the implantation, although we did not resect and histopathologically examine the graft in this case. Non-anastomotic false aneurysms in the middle portion of a vascular Dacron graft have been reported because of structural failure 12 years after femoral bypass [4]. We found no signs of suture deterioration or infection in the present case, although material fatigue is suggested as a cause of pseudoaneurysm.

Thoracic re-entry via median sternotomy carries the risk of aneurysm injury and potentially fatal hemorrhage [5]. We performed median resternotomy after exposure of the left femoral artery and vein and opened the pseudoaneurysm under CPB to avoid catastrophic bleeding. Full heparinization for CPB enabled us to detect a small bleeding and to confirm hemostasis after the repair, although an urgent start of CPB was not required in the present case. Endovascular options such as thoracic endovascular aortic repair are also possible if anatomically they are available [6]. We chose resternotomy because the bleeding point could not be detected by aortography.

Re-total arch replacement is preferable to bleeding repair because graft infection and material fatigue may not be neglected even when no signs of infection are observed. It was difficult to expose the graft completely because of adhesion, and we performed suture repair in the present case. Further, careful observation is mandatory, although the clinical course after surgery and CT showed no signs of bleeding or infection.

## Conclusion

We report a case of the pseudoaneurysm of graft-graft anastomosis 22 years after total arch replacement. Long-term follow-up is essential to detect late complications at the site of hand-sewn anastomosis.

## Consent

Written informed consent was obtained from the patients for publication of this Case report. Copies of the written consent forms are available for review by the Editor-in-Chief of this journal.

## Abbreviations

CT: computed tomography
POD: postoperative day
CPB: cardiopulmonary bypass
